# Southern hemisphere ceratosaurs evolved feeding mechanics paralleling those of Northern hemisphere tyrannosaurids

**DOI:** 10.1038/s41598-025-32686-4

**Published:** 2026-01-21

**Authors:** Andre J. Rowe, Mauricio A. Cerroni, Emily J. Rayfield

**Affiliations:** 1 School of Earth Sciences, University of Bristol, Bristol, BS8 1RJ UK; 2 Consejo Nacional de Investigaciones Científicas y Técnicas (CONICET), Buenos Aires (CABA), Argentina; 3 Área Laboratorio e Investigación, Museo Municipal “Ernesto Bachmann” (MEB), Dr. Natali S/N 8311, Villa El Chocón, Neuquén, Argentina; 4 Laboratorio de Anatomía Comparada y Evolución de los Vertebrados, Museo Argentino de Ciencias Naturales “Bernardino Rivadavia”, Buenos Aires, Argentina

**Keywords:** Ecology, Ecology, Evolution, Zoology

## Abstract

Ceratosaurs (Ceratosauria), including *Ceratosaurus* and derived abelisaurids such as *Carnotaurus*, were the dominant large-bodied predators in Cretaceous ecosystems of the Southern Hemisphere, paralleling the ecological role of tyrannosaurids in the Cretaceous Northern Hemisphere. They are known for their short, deep skulls and prominent cranial ornamentation. These traits have often been interpreted as potential adaptations for intraspecific combat, sexual display, and as a buttress during feeding-induced mechanical stresses. While these adaptations have often been discussed, they have been subject to little mechanical testing, particularly using 3D models due to previous limitations in software. Here we used a mixture of computed tomography (CT) scanning and surface scanning to create accurate 3D models of four ceratosaurian skulls: *Ceratosaurus*, *Masiakasaurus*, *Carnotaurus*, and *Majungasaurus*. Using finite element analysis, we assessed the mechanical performance of the skull during feeding, notably including the small-bodied noasaurid *Masiakasaurus*. Our results show that despite their compact shape, large abelisaurs do not exhibit low stress under bite loading. Cranial ornamentation also fails to act as a structural buttress. Additionally, functional analyses of *Masiakasaurus* combined with its procumbent teeth imply that it was suited for grasping small prey, suggesting niche partitioning with the large sympatric abelisaur *Majungasaurus*. These findings challenge prevailing assumptions about ceratosaur skull strength and suggest that large abelisaurs fulfilled ecological roles comparable to large tyrannosaurids.

**Supplementary Information:**

The online version contains supplementary material available at 10.1038/s41598-025-32686-4.

## Introduction

Ceratosauria is a taxonomically and morphologically diverse clade of basal, non-tetanuran theropod dinosaurs distributed across the Late Jurassic and Cretaceous^1^. Although theropods are broadly known for achieving large body sizes and for their ecological dominance as bipedal carnivores^2–5^, the ceratosaurs represent an under-researched evolutionary pathway within this group. Basal ceratosaurs, such as the Kimmeridgian Late Jurassic *Elaphrosaurus*, were relatively lightly built^6^, but by the Maastrichtian Late Cretaceous the group produced large-bodied predators such as *Carnotaurus sastrei*, which may have exceeded 1,700 kg in body mass^7–9^. The group is commonly associated with the Southern Hemisphere^10–12^, in contrast to tyrannosaurids which were the most prevalent carnivorous dinosaurs of the Cretaceous Northern Hemisphere^13,14^.

Unlike many other large theropods, ceratosaurs evolved a combination of unusual cranial features, including rugose ornamentation, dorsoventrally deepened snouts, and in some taxa, extremely short skulls^12^. *Carnotaurus* is commonly noted for its prominent cranial horns^15^, and *Ceratosaurus* similarly possessed distinctive horns above the eyes and a midline nasal crest^16^. *Majungasaurus* possessed tall, rugose nasals, struts within sinuses, and a horn-like projection of the frontal^17^. Various explanations have been offered for the functional significance of these traits, including intraspecific combat^15,18^ and species recognition^19^.

A notable neoceratosaur is *Masiakasaurus knopfleri*, a small-bodied noasaur from the Maastrichtian of Madagascar with a body mass under 50 kg^11,20^. Despite its relatively small size, *Masiakasaurus* possessed a highly modified skull and dentition, including forward-projecting teeth at the anterior of the jaw, possibly indicating specialized feeding behaviors and biomechanics^21^. The inclusion of *Masiakasaurus* offers an opportunity to evaluate how skull mechanics and stress distribution vary across a broad range of ceratosaur body sizes and skull morphologies, as well as the biomechanical implications of procumbent teeth in a diapsid.

**Fig. 1:**
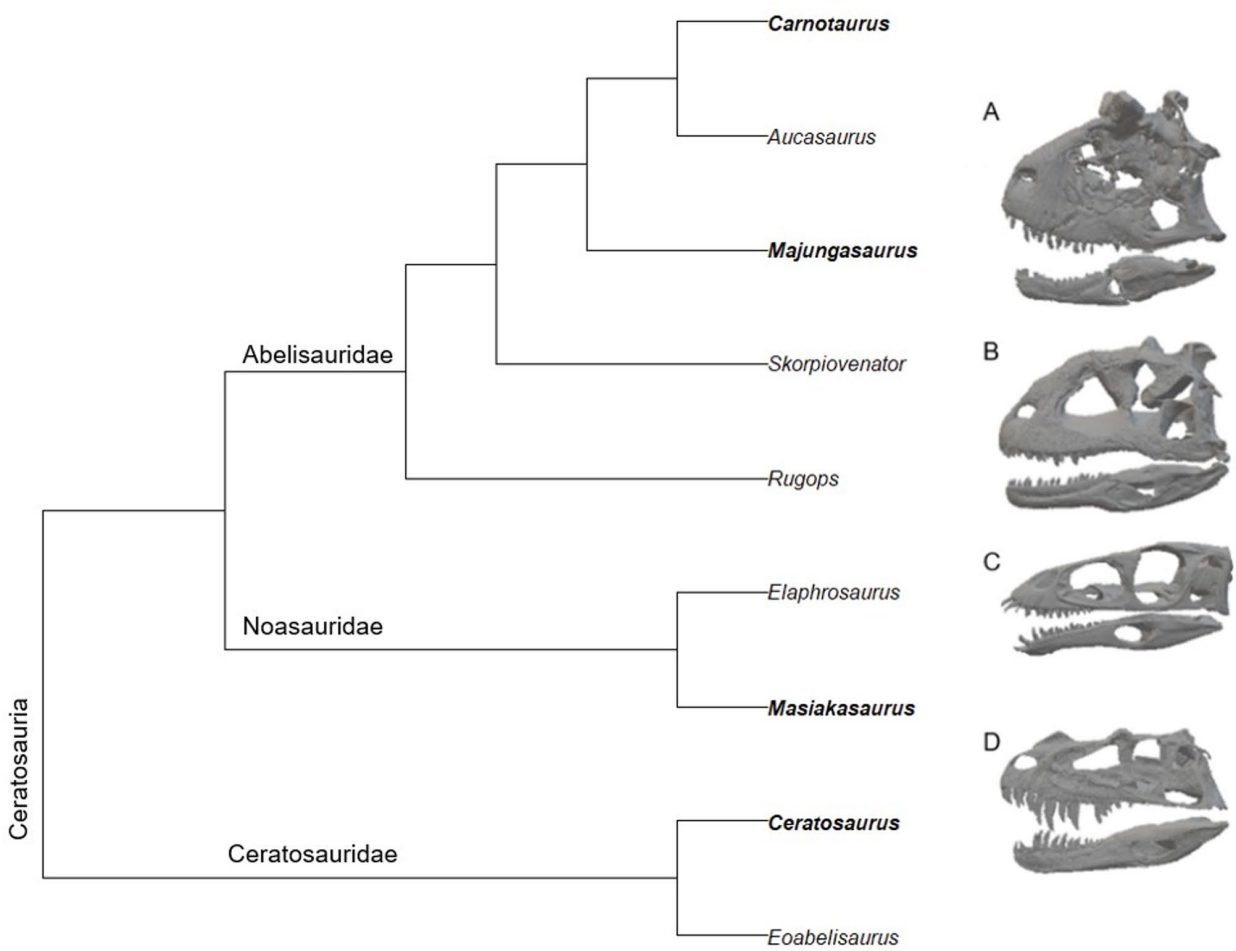
Simplified Ceratosauria cladogram based on Delcourt^12^. (**A**) *Carnotaurus*; (**B**) *Majungasaurus*; (**C**) *Masiakasaurus*; (**D**) *Ceratosaurus*. 3D models not to scale.

Body size is a key trait affecting feeding mechanics. Larger body size may facilitate higher bite forces due to greater absolute adductor muscle mass but also impose structural constraints that can influence cranial stresses^22–24^. The effects of body size on cranial function remain poorly understood in extinct clades, particularly among ceratosaurs. Large ceratosaurian taxa tend to exhibit deep, robust skulls, whereas smaller taxa display longer, more gracile morphologies (Fig. 1). These size-related changes in skull shape differ from those seen in other large theropod clades: tyrannosaurids reinforce anterior skull regions^25^, while spinosaurs elongate their rostra^26,27^, reflecting distinct biomechanical strategies associated with extreme size. Ceratosaurs were also temporally and phylogenetically distinct from other large South American theropods, including Jurassic megalosauroids such as *Asfaltovenator*^28^ and *Piatnitzkysaurus*^29^, as well as Late Cretaceous megaraptorids, e.g., *Joaquinraptor*^30^. This context highlights that the cranial modifications observed in ceratosaurs represent a lineage-specific response to increasing body size rather than a universal pattern among contemporaneous large predators.

**Fig. 2:**
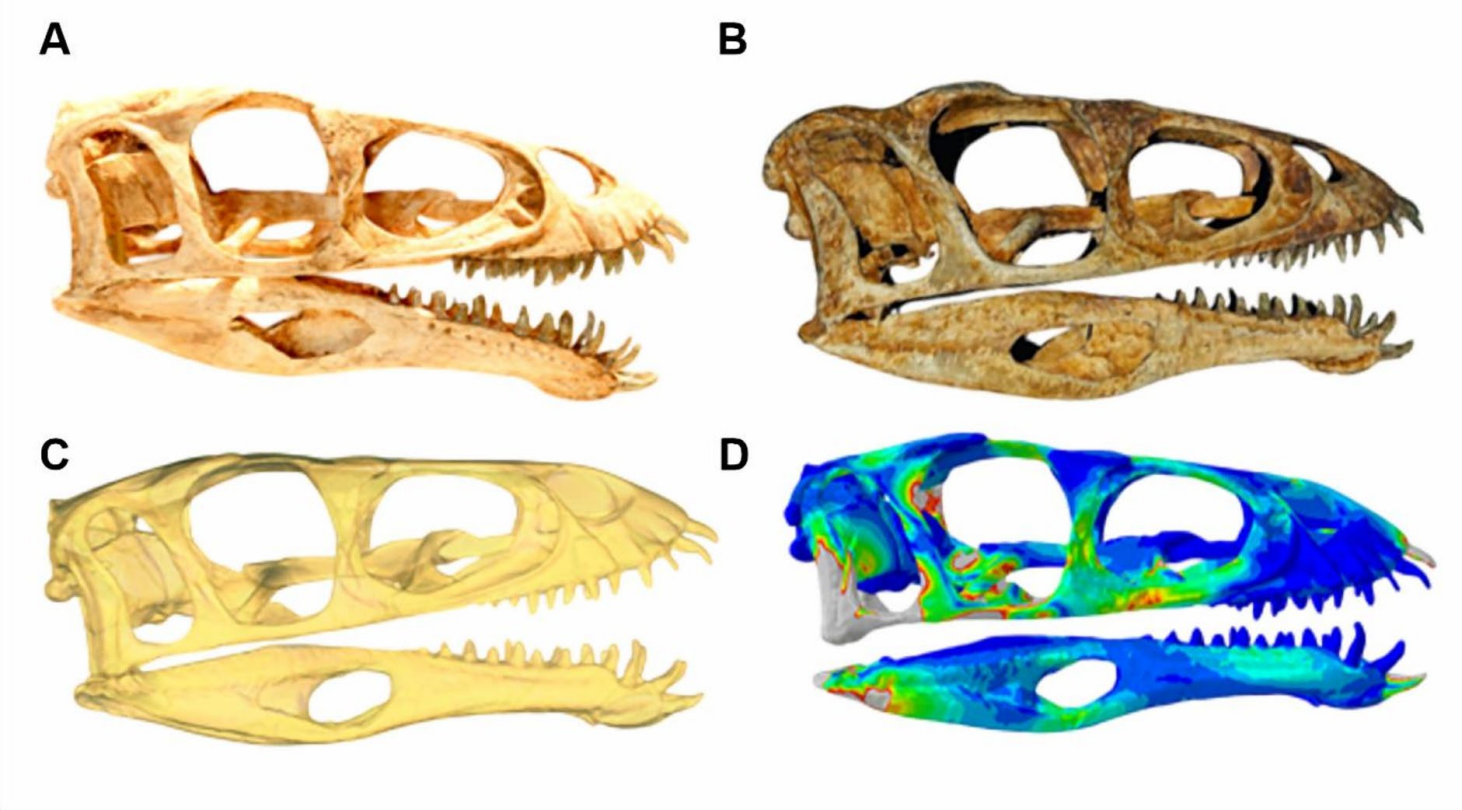
Scanning and FEA pipeline using *Masiakasaurus* replica skull. (**A**) *Masiaksaurus* skull mount at FMNH; (**B**) surface scan of *Masiakasaurus* skull in Artec Studio Professional 14; (**C**) finalized STL file of *Masiakasaurus* skull in Geomagic Wrap; (**D**) *Masiakasaurus* FE model in Abaqus/CAE.

Advances in digital modelling techniques, such as surface scanning and 3D finite element analysis (FEA), now allows for the investigation of these questions using 3D reconstructions of fossil skulls (Fig. 2)^31–34^. FEA can simulate the mechanical response of a skull to loading, providing estimates of stress and strain as proxies for structural performance. Such studies have increasingly informed our understanding of feeding adaptations in extinct taxa but have yet to focus on ceratosaurs as a lineage.

Here we apply 3D FEA to a series of ceratosaurian taxa spanning a wide range of skull sizes and morphologies: from the gracile, possibly-specialized *Masiakasaurus* to the deep-skulled, large-bodied *Carnotaurus*. Our aim is to evaluate how divergent skull morphologies influence cranial stress, mandibular stress, and feeding performance within Ceratosauria using a diverse sample of taxa.

We test the following main hypotheses:

### Hypothesis 1

Skull stress decreases as ceratosaurs increase in size due to muscle volume.

### Hypothesis 2

Skull stress remains constant or increases in large-bodied ceratosaurs due to robust skull architecture in large abelisaurs that permit greater muscle force despite increasing size.

By focusing exclusively on ceratosaurs and incorporating high-resolution digital biomechanical models, this study seeks to provide new insights into how skull and tooth anatomy interact in shaping feeding adaptations in this distinctive clade of predatory dinosaurs.

## Results

### Cranial stress patterns and plots

**Fig. 3:**
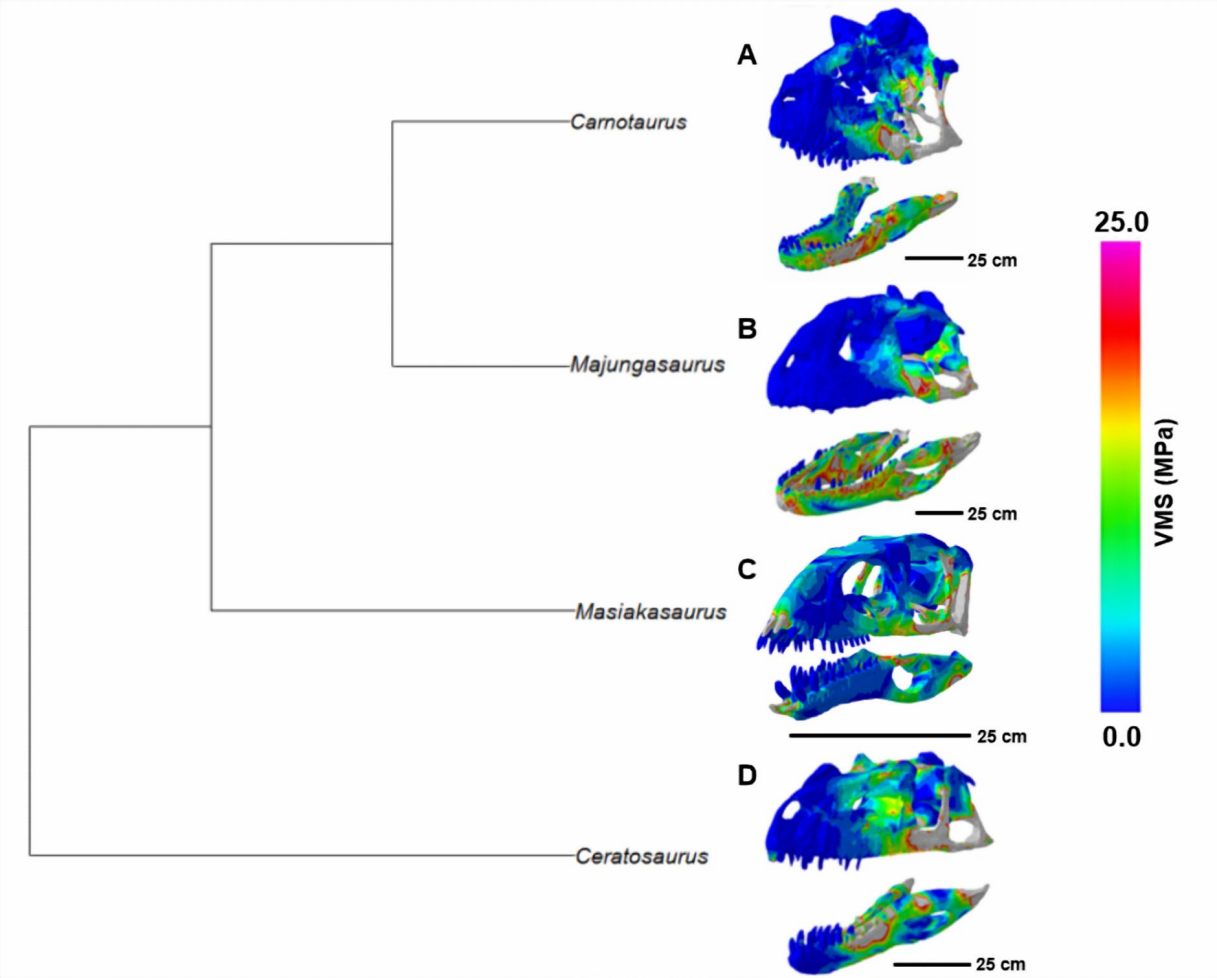
Ceratosaur FE results. (**A**) *Carnotaurus*; (**B**) *Majungasaurus*; (**C**) *Masiakasaurus*; (**D**) *Ceratosaurus*. Lower skull stresses are indicated by cooler colors, i.e., blue and green. Scale bars represent 25 cm.

**Fig. 4:**
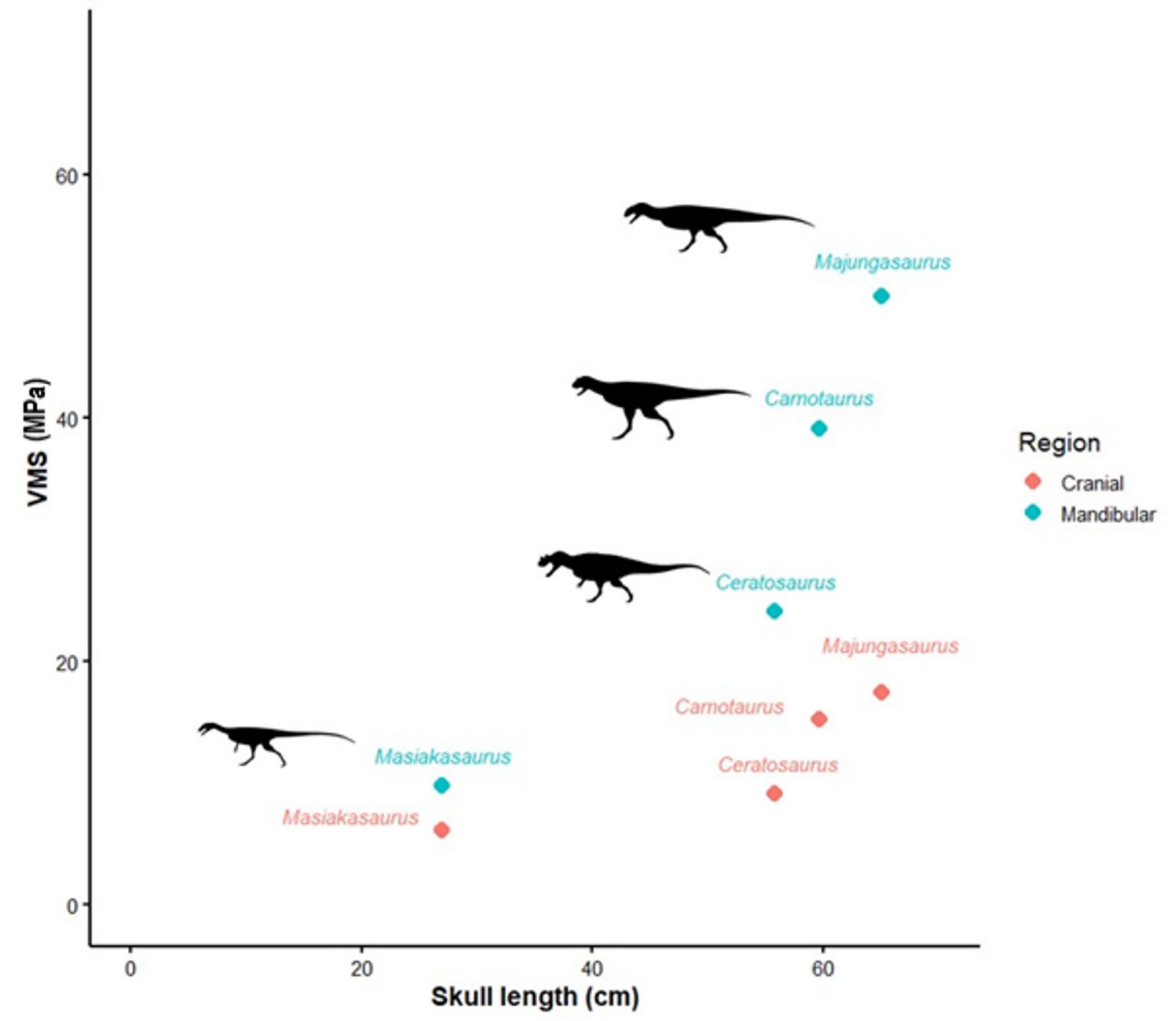
Ceratosaur skull lengths plotted against mean von Mises stress for both cranial and mandibular FE models. Silhouette images from PhyloPic by Scott Hartman.

*Majungasaurus* experiences the highest cranial stress, followed by *Carnotaurus* (Figs. 3 and 4). These stresses are most noticeable at the quadrate and quadratojugal due to the positioning of our constraints, though the stresses in *Carnotaurus* wrap forward onto the jugal. *Ceratosaurus* similarly experiences higher stresses at the jugal, though overall cranial stresses are not as high as those in the abelisaurs. *Masiakasaurus* overall experiences the lowest cranial stresses, with the stresses most noticeable at its teeth.

Cranial ornamentation appears to have little effect on absorbing cranial stresses. The horns in *Carnotaurus* are not mechanically affected by stresses and constraints applied to the quadrate and teeth; likewise, the bony struts of the frontal horn in *Majungasaurus* are unaffected. The nasal horn in *Ceratosaurus* appears to offer no functional advantage in feeding; however, the small hornlets above the orbits display moderate stresses.

### Mandible stress patterns and plots

As in the cranial results, *Majungasaurus* experienced the highest mandibular stresses, which are noticeable throughout the FE model (Fig. 3). Similarly, *Carnotaurus* experienced high mandibular stress, in particular near the anterior end of the dentary. The lower stresses experienced by *Masiakasaurus* and *Ceratosaurus* are comparable to each other. *Masiakasaurus* experienced the lowest overall stresses; the dentary appears less susceptible to breakage than other ceratosaurs in the dataset when forces are applied to the anterior teeth. Overall, there is a clear trend of increasing skull stresses with increasing skull size (Fig. 4), which is particularly notable in the two abelisaurids.

### *Masiakasaurus* biting scenarios

**Fig. 5:**
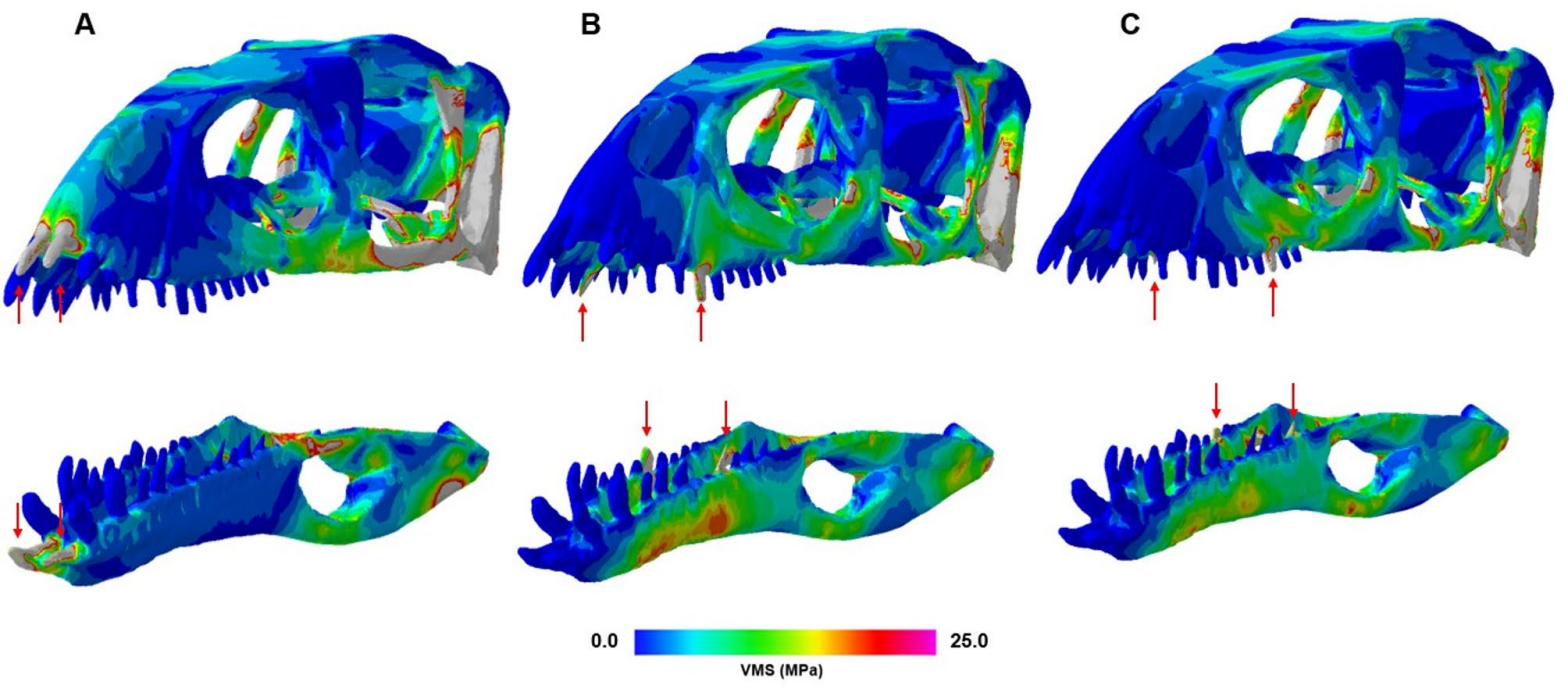
Comparison of von Mises stress distributions for different *Masiakasaurus* biting scenarios. (**A**) Forces applied to the anterior (procumbent) teeth; (**B**) forces applied to the midline teeth; (**C**) forces applied to the posterior teeth. Note the decreased stresses along the mandible in (**A**).

The small-bodied noasaur *Masiakasaurus* was further tested by applying forces to the midline teeth and posterior teeth to further elucidate the biomechanical implications of procumbent teeth (Fig. 5). We found that when forces were applied only at the front teeth, stresses acting at the mandible were decreased overall, though the quadrate and jugal are more highly stressed. Forces applied at the midline and posterior teeth resulted in much higher stresses acting on the mandible. This may be due to procumbent teeth functioning to isolate or reduce load transfer to the rest of the mandible (see Discussion).

## Discussion

Despite their distinct cranial morphologies and phylogenetic separation from other theropod lineages, ceratosaurs overall do not appear to exhibit unique biomechanical adaptations for stress resistance during feeding. Cranial ornamentation in these taxa, such as the prominent nasal horn of *Ceratosaurus*, does not appear to buttress the skull against feeding-induced stress, though the small horns above the orbits, formed by lacrimal bones, do appear stressed during feeding scenarios. Finite element analyses reveal von Mises stress magnitudes in *Ceratosaurus* that are comparable to similarly sized theropods lacking such ornamentation, suggesting that these cranial features primarily served non-feeding functions, such as species recognition or intraspecific display. The two large abelisaurs in our dataset, *Carnotaurus* and *Majunagasaurus*, appear to utilize their short, broad skulls to better absorb high feeding stresses, which is a trait shared by large tyrannosaurids.

Our results show that high von Mises stresses in ceratosaur skulls are comparable to those experienced by tyrannosaurids, as the magnitude of stresses increases in large abelisaurids, which has previously been noted in large tyrannosaurids^24,35–38^. Previous interpretations of *Carnotaurus* estimated low bite forces and rapid biting movements^15,39^. Other interpretations have argued that it possessed a relatively high bite force of ~ 7,000 Newtons^40^, and at least twice the bite force of the American alligator, which likely possesses the strongest bite of any extant tetrapod^41^.

The parallel evolution of large-bodied ceratosaurs in Gondwana and tyrannosaurids in Laurasia represents a compelling case of functional convergence within non-avian dinosaurs. Despite their disparate cranial morphologies and independent evolutionary histories, both lineages evolved skulls capable of withstanding high feeding stresses as they achieved gigantic size, implying that similar biomechanical constraints shaped apex predation in geographically isolated ecosystems. This pattern aligns with broader examples of convergent ecological expansion in theropods, such as the repeated evolution of deep, reinforced rostra in large-bodied predatory lineages and the niche partitioning documented among large tyrannosaurids and alioramins^38,42^. Within Ceratosauria itself, previous studies have noted iterative trends toward cranial deepening, reduction of forelimbs, and increasingly specialized feeding morphologies in derived abelisaurids^1,43^, paralleling structural amplifications seen in later-branching tyrannosaurids. Together with our FE results, these recurring evolutionary trajectories across multiple theropod clades suggest that the demands of subduing large prey frequently channel lineages toward similar biomechanical solutions, even when starting from contrasting anatomical templates.

Large abelisaurids including *Carnotaurus* and *Majungasaurus* appear to have occupied a biomechanical and ecological space analogous to that of large tyrannosaurids. Like in tyrannosaurids, larger abelisaur skulls appear to be more highly stressed than in ancestral ceratosaur taxa such as *Ceratosaurus*. Though the ontogeny of *Carnotaurus* and other large abelisaurs remain relatively unknown, it may be the case as in tyrannosaurids that juvenile forms of derived taxa fed similarly to their adult ancestors^37^. However, these interpretations should be considered cautiously due to the small sample size of complete abelisaur skulls suitable for 3D FE testing.

Cerroni et al.^43^. argued that the flexibility of *Carnotaurus*’s mandible may have allowed some degree of kinesis; however, the stiff cranium would prevent flexibility. Our results may reaffirm this work; *Carnotaurus* and especially *Majungasaurus* experience the highest mandible stresses, while cranial stresses were noticeably lower, particularly at the anterior of *Majungasaurus*’s cranium. In general, cranial kinesis is regarded as difficult to prove in any dinosaur species and was likely absent in most^44^. Additionally, theropod mandible stress magnitudes are almost always higher than their crania due to the size and structural complexity of animal crania compared to mandibles^24^.

Larger ceratosaurs exhibit proportionally higher von Mises stresses than smaller taxa. This pattern aligns with expectations from biomechanical scaling theory. Bite forces are estimated from the summed cross-sectional area of the adductor chamber (PCSA), which scales with the square of linear dimensions, whereas skull cross-sectional area resisting bending scales similarly; however, if skulls were perfectly geometrically similar, stress (force/area) should remain roughly constant across size. Stresses increasing with size indicate that the skulls are not geometrically similar, and that changes in shape modulate how muscle forces are transmitted. Larger abelisaurids such as *Carnotaurus* and *Majungasaurus* possess short, deep crania that are likely to improve resistance to dorsoventral bending but also concentrate stresses, while smaller taxa like *Ceratosaurus* retain elongate skulls that dissipate loads over greater distances. The biomechanical patterns in this study correspond closely with the accelerated evolutionary rates of cranial characters reported for derived abelisaurids^45^. Their results suggest that the rapid restructuring of the abelisaurid skull, particularly the shift toward short, deep, highly modified facial regions occurred in tandem with the functional changes we document, as these morphologies increasingly shaped how feeding forces were distributed in large-bodied taxa.

These findings also bear on previous interpretations of *Majungasaurus* as a “bite-and-hold” predator^17^. Such feeding behavior would impose sustained loads on the skull during prey handling and would be facilitated by a stout cranial shape capable of absorbing high stresses without structural failure. Our FE results are consistent with this: *Majungasaurus* exhibits relatively lower cranial stresses at its anterior than expected given its size, suggesting its skull was mechanically robust under sustained loading, while its mandible shows very high stresses consistent with resisting prey struggling. These findings support the functional plausibility of bite-and-hold feeding in *Majungasaurus*, though further work on muscle reconstruction and puncture mechanics would be required to test this explicitly.

*Masiakasaurus* is commonly noted for its procumbent anterior dentition, gracile skull, and relatively small body size. Unlike the typical abelisaur skull, which is short and deep, *Masiakasaurus*’s skull is long and low^20^. Our FE results support interpretations of a generalist feeding ecology that did not require powerful biting or force transmission and likely focused on small vertebrates and invertebrates, as noted in the related genus *Vespersaurus*^46^. Because procumbent teeth project forward, they direct forces more axially along their own long axes, producing lower bending moments at the alveolar margin. More posterior teeth, being upright, transmit bite forces more directly into the mandible, causing higher bending and shear stresses throughout the jaw (Fig. 5). Thus, procumbent teeth are likely specialized for low-stress prey capture or nipping, rather than for generating high bite forces. These functional interpretations also suggest predictions for isolated abelisaurid teeth, which are commonly recovered in Late Cretaceous deposits^47^. In taxa with gracile, procumbent anterior dentition such as *Masiakasaurus*, tooth morphology should reflect low bending loads, manifesting as slender crowns with reduced apicobasal wear and minimal chipping. In contrast, the more upright posterior teeth, and those of large-bodied abelisaurids generally, would be expected to show higher frequencies of wear facets, enamel spalling, or microfracturing associated with resisting greater bending and shear forces during prey handling.

Given the sympatric relationship between *Masiakasaurus* and *Majungasaurus*, it is likely that noasaurs maintained small, slender physiologies into maturity to avoid competition with large abelisaurs. Niche partitioning has been noted in other theropod clades previously, including the alioramins and their larger relatives, e.g., *Alioramus* and *Tarbosaurus*^38,42^. However, the procumbent teeth which are characteristic of *Masiakasaurus* have not been described in any tyrannosaurid taxa; this may suggest *Masiakasaurus* utilized these teeth to effectively grasp food items in nutrient or resource-poor environments of Late Cretaceous Madagascar.

## Conclusion

Ceratosaurs display a range of biomechanical adaptations to feeding efficiency. As body size increases, abelisaur skulls exhibit higher absolute von Mises stresses, paralleling the pattern observed in tyrannosaurids, despite their independent evolutionary histories and distinct cranial morphologies. This suggests that both lineages experienced similar biomechanical constraints at gigantic size. In large abelisaurs such as *Majungasaurus*, elevated stresses coincide with cranial shapes that appear well suited to resisting feeding-induced loads, supporting interpretations of relatively high bite forces and possibly a “bite-and-hold” feeding strategy. The small noasaur *Masiakasaurus*, in contrast, appears to have used its procumbent teeth and low force transmission to capture small vertebrates, invertebrates, or other available resources. *Ceratosaurus* may have derived minor cranial support from its lacrimal horns, though its prominent nasal horn likely functioned primarily in display. Overall, our results indicate that abelisaurs converged on the biomechanical and ecological roles occupied by tyrannosaurids in the Northern Hemisphere, with both clades evolving skulls capable of tolerating high feeding stresses as they achieved large size.

## Materials and methods

### Institutional abbreviations

**BYUVP**: Brigham Young University Museum of Paleontology; **DDM**: Dinosaur Discovery Museum; **FMNH**: Field Museum of Natural History; **MACN-Pv**: Museo Argentino de Ciencias Naturales “Bernardino Rivadavia”; **UA**: University of Antananarivo, Madagascar.

### Ceratosaur taxonomy and sampling

The earliest ceratosaur representative, *Saltriovenator*, dates to the Sinemurian Early Jurassic of Italy (199 Ma)^48^. The clade survived to the Maastrichtian end Cretaceous (66 Ma), where the Madagascan taxa *Majungasaurus* and *Masiakasaurus* have been recovered^49^. Ceratosaurs are generally recognized for their robust, highly ornamented yet short skulls and extremely reduced forelimbs. They are primarily found in the Southern Hemisphere though not exclusively^12,50^. *Ceratosaurus* is the most primitive taxon examined in this study and is characterized by its prominent, thin nasal horn which may have functioned for display and/or intraspecific combat^51–53^, as well as unusually fast growth^54^ and elongated, irregularly formed osteoderms along the midline of its body^55^.

Previous biomechanical studies concerning *Carnotaurus* have deemed it capable of quick but relatively weak bites, which are critical for capturing small prey^15,39^. Therrien et al.^41^. argued that *Carnotaurus* possessed a bite force twice that of the American alligator, which has one of the highest bite forces in extant species, and it would have been adept at delivering slashing wounds to large prey. Novas^18^ argued that the well-developed postorbital flanges encircling the orbit in *Carnotaurus* may have dimmed potential wounds to the eye and head, whereas Cerroni et al.^43^. assessed the potential flexibility of the skull and determined that any flexibility would be limited to the mandible, as the thickened skull roof and ossification of several cranial joints suggests little or no cranial kinesis.

Noasaur feeding biology has remained largely understudied due to lack of skull material. Barbosa et al.^46^. examined an isolated tooth and two pedal unguals of *Vespersaurus paranaensis* using 3D FEA and concluded it would have functioned as a generalist feeder preferring small prey and carcasses. This is the first biomechanical study of a 3D noasaur skull. *Ceratosaurus*, *Masiakasaurus*,* Carnotaurus*, and *Majungasaurus* were included in this study (Fig. 1; Table 1).

**Table 1:** Skull lengths and body mass estimates for each taxon. Skull lengths were measured in MeshLab 2022.02 and masses were referenced from the literature.

Specimen name	Specimen number	Scanning method	Skull length (cm)	Body mass estimate (kg)	Body mass source
*Ceratosaurus nasicornis*	BYUVP 12,893	Surface scan	55.8	418	Therrien & Henderson^8^
*Masiakasaurus* *knopfleri*	Composite based on UA 8680 and FMNH PR 2183	Surface scan	27	33–47	Carrano et al.^22^
*Majungasaurus crenatissimus*	FMNH PR 2100	Surface scan	65	1,130	Sampson & Witmer^17^
*Carnotaurus sastrei*	MACN-Pv-CH 894	CT scan	59.6	1,350	Bonaparte et al.^7^

### 3D imaging

We measured skull lengths in MeshLab 2022.02 to better understand possible relationships between size and skull stresses. 3D models originated from both computed tomography (CT) scanning and blue light surface scanning; *Carnotaurus* is a CT-derived model of the original fossil, while the rest are replicas (Table 1; Table S1).

The *Masiakasaurus* skull used in this study is based on a composite cast housed at FMNH. The original fossil material comprises a partial right maxilla (FMNH PR 2183) and a partial dentary (UA 8680). All remaining cranial and mandibular regions of the cast were reconstructed to restore overall skull shape, based on mirrored copies of preserved elements and comparative morphology from closely related noasaurids described by Sampson et al.^20^. ; hence, the model is a hypothesis of what a complete, non-deformed specimen would resemble.

The *Carnotaurus* model was segmented from CT data based on the original skull material (MACN-Pv 894). The holotype was CT-scanned using a CT 64 Ingenuity Core medical tomographer, with the skull and left jaw scanned separately. The slice thickness of both scans was about 0.62 mm and scan energy parameters for the skull were 119 mA and 120 kV, and for the lower jaw were 79 mA and 120 kV.

*Ceratosaurus* was surface scanned at the DDM. The DDM cast is based on BYU specimen 12,893, supplemented with comparative material from the larger *Ceratosaurus* specimens at the Smithsonian National Museum of Natural History and Cleveland Museum of Natural History to complete missing elements; however, the skull material is entirely based on the BYU specimen. The *Majungasaurus* replica was scanned at the FMNH. It is composed of the cranium with a detached mandible, which made surface scanning easier as the mandible did not obscure the ventral portion of the cranium.

### Model editing

Surface scanned models were created in Artec Studio 14 Professional. The scans were captured at 7–8 frames per second, with the ‘real-time fusion’ option enabled. Crania and mandibles were all scanned separately whenever possible and created as separate 3D object files to avoid large file sizes, since these can cause software slowdown or crashes. Surface scans were oriented together by selecting coordinates and then merging into a single object. Stray pixels and frames with maximum error values above 0.3 were deleted. We applied Global Registration to convert all one-frame surfaces to a single coordinate system using information on the mutual position of each surface pair and then selected ‘Sharp Fusion’ to create a polygonal 3D model, which solidifies the captured and processed frames into an STL file. ‘Sharp Fusion’ best preserves fine details of the scans, including small teeth and rugose bone textures which are particularly common in abelisauroids like *Majungasaurus*. Lastly, we used the small-object filter to clean the model of floating pixels and the fix holes function to fill any open areas, as the model must be watertight for 3D meshing to proceed without errors (Fig. 2).

Once all models were finalized in Geomagic Studio 12, the model surface areas, numbers of triangles, elements, and the volume were calculated and recorded for each model (Table S1, Table S2).

### Finite element analyses

All models were meshed using HyperMesh (Altair) software (https://altair.com/hypermesh). Element size was set to 10 mm, and in the Tetramesh parameters sub-panel, we selected for optimized mesh quality and for the mesh speed to be gradual. Once the model was meshed successfully, we applied the appropriate material properties to the meshed models. The bone properties were assigned based on crocodilian skull bone: Young’s modulus of 15,000 MPa and Poisson’s ratio of 0.29^56,57^ for each theropod dinosaur given the close evolutionary relationship between crocodilians and dinosaurs. No sutures were modeled; dentine properties were also not modeled, as they contribute minimally to stress distribution in comparative analyses^58^.

Six total constraint nodes were applied to the quadrate of each cranium and the articular of each mandible to prevent rigid body motion of the models during loading. To achieve this, one node at each location was constrained in a single degree of freedom (DOF) along the X, Y, or Z axis, collectively eliminating translation and rotation while still allowing deformation. Three additional constraint nodes were applied to two premaxillary teeth on each side of the cranium and mandible to simulate contact with prey during a bite. For *Masiakasaurus*, we additionally conducted FE tests in which bite forces were applied separately to the midline, and posterior teeth to assess how bite point location influenced stress distribution across the skull and elucidate the possible impact of procumbent teeth. Once the mesh was imported into Abaqus/CAE (https://www.3ds.com/products/simulia/abaqus/cae), we applied adductor muscle forces to the models to accurately assess the effects of muscle loading on the skull during a feeding simulation. Locations of muscle insertions were approximated from Holliday^59^, Rowe & Snively^35^, and direct study of the specimens. Muscle forces for all theropods were scaled from adult *Tyrannosaurus* FMNH PR 2081 using the subtemporal fenestra method outlined in Sakamoto^60^ (Table S3). This was done by measuring the dimensions of the subtemporal fenestra using ImageJ and multiplying the surface area by the isometric muscle tension of 31.5 N/cm^2^. This method functions as a reliable proxy across amniote clades, and no theropod dinosaur taxa appear to be outliers^61^.

We generated von Mises stress data for each theropod dinosaur cranial and mandibular model. 3D finite element stress maps were generated for each 3D model analyzed, with von Mises stress limits ranging from 0 to 25 MegaPascals in the display figures. Mesh-weighted arithmetic mean (MWAM) values were calculated using RStudio for each theropod specimen to reduce possible discrepancies between CT-derived models and surface scan-derived models and account for element volume differences between models^62–64^. Rowe & Rayfield^34^ concluded that 3D FE models with a high degree of editing applied prior to meshing tend to output relatively high von Mises stress and maximum principal strain relative to un-modified models. Mesh-weighted von Mises stress data was compiled and compared for all skulls in a phylogenetic context to visualize the effects of large skulls on feeding-induced stresses (Fig. 3). Given that our dataset includes only four taxa, formal phylogenetic generalized least squares (PGLS) regression was not attempted, as such analyzes are statistically underpowered and would yield unstable estimates of phylogenetic signal. Instead, we present von Mises stress values quantitatively and descriptively within a phylogenetic framework.

## Supplementary Information

Below is the link to the electronic supplementary material.

Supplementary Material 1

## Data Availability

FE files available upon request from the corresponding author, Andre J. Rowe (andre.rowe@bristol.ac.uk).
