# Supplementary material for: Southern hemisphere ceratosaurs evolved feeding mechanics paralleling those of Northern hemisphere tyrannosaurids
Source: Sci Rep. 2026 Jan 21;16:2804. doi: 10.1038/s41598-025-32686-4 (PMC12824262; doi:10.1038/s41598-025-32686-4)
Supplement: Supplementary file 1 — Supplementary Material 1 [file 41598_2025_32686_MOESM1_ESM.docx]

**Table S1. 3D models, number of triangles, elements, and volume measurements (mm^3^).**

| Specimen | Cranial triangle count | Mandibular triangle count | Cranial element count | Mandibular element count | Cranial volume (mm³) | Mandibular volume (mm³) |
| --- | --- | --- | --- | --- | --- | --- |
| *Ceratosaurus nasicornis* | 398,798 | 314,918 | 598,197 | 472,377 | 199,391 | 157,459 |
| *Masiakasaurus*  *knopfleri* | 350,006 | 315,016 | 525,009 | 472,524 | 174,985 | 157,506 |
| *Majungasaurus crenatissimus* | 498,980 | 399,982 | 748,470 | 599,793 | 249,472 | 199,898 |
| *Carnotaurus sastrei* | 497,700 | 398,508 | 746,550 | 597,762 | 248,756 | 199,249 |

**Table S2. Surface area values (mm^2^) for the cranial and mandibular models of each taxon.**

| Specimen name | Cranial model surface area (mm^2^) | Mandible model surface area (mm^2^) | Replica of skull? |
| --- | --- | --- | --- |
| *Ceratosaurus nasicornis* | 360584 | 182932 | Yes |
| *Masiakasaurus*  *knopfleri* | 24455 | 9898 | Yes |
| *Majungasaurus crenatissimus* | 706964 | 258754 | Yes |
| *Carnotaurus sastrei* | 949904 | 306760 | No |

**Table S3. Muscle forces used in actual size ceratosaur skull analyses in newtons (N).**

| *Ceratosaurus* | mames | mamem | mamep | mamp | mps | mptd | mptv |
| --- | --- | --- | --- | --- | --- | --- | --- |
| Left mandible ramus/cranium half |  |  |  |  |  |  |  |
| x | 5082.639 | 1909.964 | 4788.051 | 4003.393 | 3980.307 | 1035.347 | 16041.84 |
| y | -236.751 | -50.0004 | 188.9123 | 272.9724 | 1188.179 | 146.7919 | 5852.755 |
| z | 1625.201 | 503.6992 | 992.0314 | 1011.129 | 1230.809 | 1072.629 | -6089.19 |
| Right mandible ramus/cranium half |  |  |  |  |  |  |  |
| x | -5082.64 | -1909.96 | -4788.05 | -4003.39 | -3980.31 | -1035.35 | -16041.8 |
| y | -236.751 | -50.0004 | 188.9123 | 272.9724 | 1188.179 | 146.7919 | 5852.755 |
| z | 1625.201 | 503.6992 | 992.0314 | 1011.129 | 1230.809 | 1072.629 | -6089.19 |
| *Masiakasaurus* | mames | mamem | mamep | mamp | mps | mptd | mptv |
| Left mandible ramus/cranium half |  |  |  |  |  |  |  |
| x | 23.52692 | 6.499186 | 8.599735 | 11.86672 | 14.58083 | 24.8469 | 20.0174 |
| y | -5.15657 | 0.989413 | -2.27151 | -1.73805 | -4.50606 | -8.92871 | -3.9655 |
| z | 111.4031 | 41.62471 | 103.7449 | 87.14832 | 105.4345 | 57.05736 | 24.34593 |
| Right mandible ramus/cranium half |  |  |  |  |  |  |  |
| x | -23.5269 | -6.49919 | -8.59974 | -11.8667 | -14.5808 | -24.8469 | -20.0174 |
| y | 4.544225 | 1.030004 | 2.23333 | 1.738053 | 4.897892 | 7.916172 | 4.069853 |
| z | 111.4031 | 41.62471 | 103.7449 | 87.14832 | 105.4345 | 57.05736 | 24.34593 |
| *Majungasaurus* | mames | mamem | mamep | mamp | mps | mptd | mptv |
| Left mandible ramus/cranium half |  |  |  |  |  |  |  |
| x | 5586.324 | 2099.239 | 5262.543 | 4400.126 | 4374.752 | 1137.949 | 17631.57 |
| y | -260.213 | -54.9554 | 207.6334 | 300.0237 | 1305.926 | 161.3388 | 6432.758 |
| z | 1786.257 | 553.6153 | 1090.341 | 1111.33 | 1352.781 | 1178.926 | -6692.62 |
| Right mandible ramus/cranium half |  |  |  |  |  |  |  |
| x | -5586.32 | -2099.24 | -5262.54 | -4400.13 | -4374.75 | -1137.95 | -17631.6 |
| y | -260.213 | -54.9554 | 207.6334 | 300.0237 | 1305.926 | 161.3388 | 6432.758 |
| z | 1786.257 | 553.6153 | 1090.341 | 1111.33 | 1352.781 | 1178.926 | -6692.62 |
| *Carnotaurus* | mames | mamem | mamep | mamp | mps | mptd | mptv |
| Left mandible ramus/cranium half |  |  |  |  |  |  |  |
| x | 8379.486 | 3148.859 | 7893.814 | 6600.189 | 6562.128 | 1706.924 | 26447.36 |
| y | -390.319 | -82.4331 | 311.4501 | 450.0356 | 1958.889 | 242.0083 | 9649.137 |
| z | 2679.385 | 830.4229 | 1635.511 | 1666.996 | 2029.172 | 1768.389 | -10038.9 |
| Right mandible ramus/cranium half |  |  |  |  |  |  |  |
| x | -8379.49 | -3148.86 | -7893.81 | -6600.19 | -6562.13 | -1706.92 | -26447.4 |
| y | -390.319 | -82.4331 | 311.4501 | 450.0356 | 1958.889 | 242.0083 | 9649.137 |
| z | 2679.385 | 830.4229 | 1635.511 | 1666.996 | 2029.172 | 1768.389 | -10038.9 |
